# Repetitive Transcranial Magnetic Stimulation (rTMS) Improves the Gait Disorders of Rats Under Simulated Microgravity Conditions Associated With the Regulation of Motor Cortex

**DOI:** 10.3389/fphys.2021.587515

**Published:** 2021-02-04

**Authors:** Jiajia Yang, Rong Liang, Ling Wang, Chenguang Zheng, Xi Xiao, Dong Ming

**Affiliations:** ^1^Institute of Medical Engineering and Translational Medicine, Tianjin University, Tianjin, China; ^2^School of Precision Instrument and Opto-Electronics Engineering, Tianjin University, Tianjin, China; ^3^Tianjin Key Laboratory of Brain Science and Neural Engineering, Tianjin University, Tianjin, China

**Keywords:** simulated microgravity, repetitive transcranial magnetic stimulation, local field potential, IGF-1-PI3K-Akt-mTOR signaling pathway, gait disorders

## Abstract

In previous studies, it has been proved that repetitive transcranial magnetic stimulation (rTMS) improves dyskinesia induced by conditions such as spinal cord injury, Parkinson diseases and cerebral ischemia. However, it is still unknown whether it can be used as a countermeasure for gait disorders in astronauts during space flight. In this study, we evaluated the effects of rTMS on the rat gait function under simulated microgravity (SM) conditions. The SM procedure continued for consecutive 21 days in male Wistar rats. Meanwhile, the high-frequency rTMS (10 Hz) was applied for 14 days from the eighth day of SM procedure. The behavioral results showed that SM could cause gait disorders such as decreased walking ability and contralateral limb imbalance in rats, which could be reversed by rTMS. Furthermore, rTMS affected the neural oscillations of motor cortex, enhancing in δ (2–4 Hz) band, suppressing in θ (4–7 Hz), and α (7–12 Hz) bands. Additionally, rTMS could activate mTOR in the motor cortex. These data suggests that the improvement effects of rTMS on gait disorders in rats under SM conditions might be associated with its regulation on neural oscillations in the cerebral motor cortex and the expression of some motor-related proteins which may enhance the control of nervous system on muscle function. Based on our results, rTMS can be used as an potential effective supplement in the field of clinical and rehabilitation research to reduce gait disorders caused by the space environment.

## Introduction

Gait disorder is one of the most common motor complications in aerospace special environment, especially microgravity (Bloomberg and Mulavara, 2003; Reschke and Clément, 2018). In this regard, the current effective measures of prevention and treatment remain to be further explored (Hodkinson et al., 2017). Repetitive transcranial magnetic stimulation (rTMS) is considered to improve dyskinesia caused by diseases such as Parkinson diseases, spinal cord injury and cerebral ischemia (Ba et al., 2016; Li et al., 2016; Arfani et al., 2017; Petrosyan et al., 2017), which transmits electromagnetic pulse signal through the skull to the cerebral cortex (Henderson et al., 2012) and improves the responsiveness of motor system by increasing the excitability of corticospinal system to achieve therapeutic purposes (Jooss et al., 2019; Khedr et al., 2019; Yang et al., 2019). The dyskinesia caused by Parkinson’s disease and spinal cord injury is mostly due to functional deficits in cortical circuits or cerebellum, or related to the peripheral nervous systems such as impaired afferent or efferent nerves (Ni and Chen, 2015; Capogrosso et al., 2016; Wirz and van Hedel, 2018; Yoo et al., 2019). However, gait disorders caused by microgravity are mostly due to muscle weakness caused by disuse atrophy and sensorimotor deconditioning caused by altered muscular sensation, which are different from the underlying causes of dyskinesia caused by the Parkinson’s disease and spinal cord injury (Colaianni et al., 2017; Bettis et al., 2018). However, it is unclear whether rTMS could also improve gait disorders by enhancing the control of the neuromuscular system on hind limbs.

Utilizing functional MRI investigation of brain activity, it was found that alteration of brain function in the astronaut was induced by long-duration spaceflight (Cassady et al., 2016; Kramer et al., 2020). Specifically, the resting state connectivity decreases were identified for the right insula as well as between the left cerebellum and the right motor cortex (Demertzi et al., 2016). The results from simulated weightlessness experiment on the ground for 45 days – 6°head down bed rest (HDBR), are consistent with the above findings (Koppelmans et al., 2017; Yuan et al., 2018). Additionally, after 70 days of HDBR, the volume of gray matter (GM) in the posterior parietal lobe was generally increased; recovery was not yet complete by 12 days post-HDBR (Koppelmans et al., 2017). In the rodent model, 14 days of tail suspension showed that the dendritic spines in the motor cortex of the rats remodeled (Trinel et al., 2013). Twenty-one-day simulated microgravity (SM) could reduce brain-derived neurotrophic factor and induce cerebral cortical neuron atrophy (Chen B. et al., 2019). These findings indicate the brain structure and function related to motor function could be affected in the real or simulated aerospace environment, including humans or rodents.

As we mentioned above, rTMS has been shown to alleviate the dyskinesia of Parkinson’s patients and the mechanism was related to regulation of neural oscillations in the cortical-basal ganglia pathway (Gaynor et al., 2008). Neural oscillations are thought to be an essential mechanism that enables the coordination of neural activity in normal brain functioning (Fuggetta and Noh, 2013). Current research has gradually identified the basic role of abnormal neural oscillations in the delta (δ) and theta (θ), and beta (β) and gamma (γ) frequency ranges observed in patients to explain the pathophysiology of dyskinesia caused by neuropsychiatric disorders (Huang et al., 2005). So we asked whether rTMS could improve the muscle control of motor cortex by regulating neural oscillations under SM conditions. This question raises profound research about the potential relationship between the effects of rTMS on the output of the motor cortex and the recovery of the controlled muscles. The goal of this study is to better understand how rTMS works, from neural oscillations to muscle control, and ultimately to apparent improvement in motor behaviors. The results will contribute to a deeper understanding of the possible electrophysiological and molecular mechanisms underlying the effects of rTMS on the brain.

In this study we found that after 2-week rTMS treatment, gait disorders caused by SM were improved to a certain extent. The mechanism was associated with the regulation of the energy distribution of different frequency bands (δ, θ, and α) of motor cortex, as well as the activation of mTOR protein by rTMS, which could enhance the control of nervous system on hindlimb muscle, ultimately achieving the improvement in gait behaviors. This provides a theoretical basis for rTMS to improve the gait disorders and proposes new treatment idea for astronaut gait disorder caused by complex space environment.

## Materials and Methods

### Animals and Ethical Approval

Male Wistar rats (180–220 g) were purchased from Beijing Vital River Laboratory Animal Technology Co., Ltd., and were used for behavioral experiments (open-field, swimming, gait analysis), electrophysiological recording, western blot, and HE staining analysis. The rats were housed individually in the animal room and were allowed to acclimate to the environment of the animal room for 7 days before the onset of each experiment. All animals were housed under normal light-dark (23°C, 12-h light/12-h dark cycle) with food and water *ad libitum*. All animal experimental protocols were approved by the Animal Management Rules of the Ministry of Health of the People’s Republic of China and the local Ethical Committee of the Tianjin University. All efforts were made to minimize the number of animals and their suffering. Rats were randomly divided into control (CON group, *n* = 10), SM for 3 weeks with sham stimulation for 2 weeks (SM+sham group, *n* = 7) and SM for 3 weeks with rTMS for 2 weeks (SM+rTMS group, *n* = 8).

### Simulated Microgravity With Sham Stimulation Rat Model and rTMS Treatment

The SM+sham rat model was built for 3 weeks, in which rats were suspended at the tail of 30 degrees and were sham-stimulated from the second week. Thirty degrees tail-suspension (TS) rats were used as the model to simulate the physiological effects of weightlessness. It was obtained using the model adapted from Wronski and Morey-Holton (Morey-Holton and Globus, 2002; Zhai et al., 2020). Briefly, each rat was first fixed with a rat-fixing device, only its tail was exposed and washed with warm water and soapy water. Then wiped with a towel as much as possible to prevent excessive stimulation of the sticker on its tail. Next, applied rosin and benzoin powder. The purpose of applying rosin powder was to make the tail astringent and better stick medical adhesive tape. And the purpose of applying benzoin powder was to prevent rats from infection and decay of tail due to long modeling time. Thirdly, the tail of the rat was wrapped in a breathable gauze, and then the nylon rope was connected with the horizontal beam of the tail suspension cage with a key clasp. The tail suspension cage is a cube of 35 × 35 × 35 cm. And the key buckle can make the rats rotate along 360 degrees horizontally to feed freely and drink water, then we can adjust the height of the horizontal beam so that the hind limbs of the rats cannot touch the bottom of the tail suspension cage when stretching, and then keep the level of the trunk and the bottom of the tail suspension cage at 30 degrees. After 21 days of SM+sham modeling, the gauze should be carefully removed for subsequent experiments.

In SM+rTMS group, we started whole brain rTMS from the second week of modeling, and rats were placed in rat fixators. The magnetic stimulator from Institute of Electrical Engineering, Chinese Academy of Sciences was used. The parameter of the magnetic stimulations were a coil with the frequency of 10 Hz, the stimulus intensity was 1T, the voltage was 800 V, each sequence had 30 pulses, there were 10 pulses in the interval of 50 s, once a day for 14 days (Wassermann and Lisanby, 2001).

### Behavioral Assessment

#### Open-Field Experiment

The open-field experiment was used to measure anxiety-like behavior and locomotor activity in an open field arena (Kraeuter et al., 2019). In this experiment, it was evaluated to the gait indicators of rats in open space with fewer interference factors, such as walking speed, walking distance, rest time in the open box, and so on. The experimental device is a cube (length: 100 cm; width: 100 cm; height: 40 cm) with black bottom. A camera is installed 70.6 cm away from the bottom to collect the rat’s trajectory. The device is surrounded by a shade. During the experiment, it is in a dark and quiet state, avoiding unnecessary influence on the experimental animals, to affect the experimental results. On a given trial, the rats were placed in the center of the apparatus. Then rats were allowed to explore freely 5 min before being removed. Video tracking software was used to collect the walking distance, speed, and other indicators of rats in the open field (Li et al., 2006; Mysoet et al., 2017). A trial was conducted in the experiment. After each session, the apparatus and wire cups were thoroughly cleaned with 75% ethanol and water to avoid affecting the next set of experiments (Xu et al., 2019).

#### Swimming Experiment

The apparatus consists of a 115-cm diameter circular pool filled with water (temperature = 23 ± 2°C) to a level of 42 cm. A visible platform (diameter = 7 cm) with a texture that allows rats to cling on it is positioned in the middle of the pool. The platform emerges ∼2–3 cm. This height makes an effort from the rats to climb on it (Canu et al., 2007). In turn, the rats were put into the water from the same position facing the wall of the pond. If the rat did not reach the platform after 60 s, it would be manually guided to the platform by the experimenter, recording the time of climbing the platform (from the beginning of reaching the platform with the forelimbs to the end of climbing all the hind limbs on the platform). Video tracking software was used to record swimming speed, swimming distance, time of immobility in water, and other parameters of rats. There was only one trial in the experiment. Only successful trials were taken into account.

#### Gait Analysis Experiment

The device is a cuboid with a runway, which length is about 63 cm and has an adjustable width. When the experimental animal is a rat, the width is generally about 10 cm. The imported cold light source 640 × 480, 120 fps, 1/4 CCD high-speed camera at the bottom of the device is used to shoot rat footprints. This device is a closed pedestrian platform. It can accurately evaluate the animal’s footsteps and gaits without any coercive measures, to obtain the natural gait. The experiment should be conducted in darkness and quiet, to reduce the interference of the environment on the experiment itself. The green fluorescent footprints can capture real and dynamic footprints by using the bright refraction technology of footprints, and the pressure of footprints can be obtained by measuring the distribution of animal weight. Gait analysis software was used to collect parameters such as footprint area, walking speed, stride length, pressure, and the relationship between footprints (Mysoet et al., 2017). After each session, the apparatus and wire cups were thoroughly cleaned with 75% ethanol and water to avoid affecting the next set of experiments.

### Electrophysiological Recording

The electrophysiological experiment was conducted to record local field potential (LFP) signals to observe the changes of nerve oscillation in the motor cortex of rats after doing behavioral environments. Briefly, rats were anesthetized with isoflurane for 3 ± 2 min, and then intraperitoneally injected with a 30% uratan solution according to 0.35% rat’s body weight. Next, the rat’s head was fixed on the stereotaxis apparatus and then removed the hair from the head. Furthermore, the rat’s eye and its surroundings were also smeared with erythromycin eye ointment and the scalp was wiped with iodine wine to prevent infection. And then we should label Primary motor cortex (M1), Secondary motor cortex (M2), and anterior fontanel according to the 6th Edition of Stereotaxic Map of Rat Brain. Among them, the coordinates of M1 and M2 area are respectively: M1: anterior fontanel 1.00 ± 0.1 mm, sagittal suture 2.00 ± 0.1 mm, depth 0.55 ± 0.1 mm; M2: anterior fontanel 3.0 ± 0.1 mm, sagittal suture 1.6 ± 0.1 mm, depth 0.4 ± 0.1 mm. The holes of about 2 mm in diameter were drilled in M1 and M2 area by skull drill, and the lower electrodes were drilled according to the experimental requirements (Figure 4B). The electrodes used in this experiment are copper electrodes with a diameter of about 0.36 mm. The LFP signal recorded by the AM system (A-M Systems, United States) was stored in a computer for subsequent analysis by MATLAB (Mathworks, United States). The recording parameters of the LFP signal are as follows: sampling frequency is 1 kHz and the recording time is 10 min.

### HE Staining

HE staining was used to exam the morphological changes of neurons in the rat motor cortex. After perfusing with PBS solution, the motor cortex was taken out. The motor cortex from left brain was used for HE staining, the motor cortex from right brain was used for the western blot. The tissue for HE staining was put into 4% polyformaldehyde solution overnight, then gradient dehydration of sucrose solution with 10 and 20% mass fraction was carried out to make the tissue sink to the bottom. Using a freezing microtome (Leica, Germany) to slice at −20°C and then slices of 15 μm thick were placed under a microscope for observation.

### Western Blot

The motor cortex was lysed RIPA lysis buffer and centrifuged for about 20 min with a centrifuge (Eppendorf, Germany) with a rotating speed of 12,000 r/min. After quantification using the BCA kit (Solarbio, United States), 50 μg of protein samples were separated by SDS–polyacrylamide gel electrophoresis under 120 V for 90 min and were transferred to PVDF membrane at 100 V for 90 min, blocked with 5% non-fat milk for 60 min. And then incubated with primary antibodies overnight at 4°C. The membrane was washed and incubated in secondary antibody for 40 min, which is Rabbit lgG antibody (HRP) (1:2000). β-actin was used as a loading control. Finally, the protein strips were exposed using an automatic light imaging system (Tanon, China). The integrated gray value of each protein band was measured by Photoshop CC 2017 (Adobe, United States).

### Quantification and Statistical Analysis

All data were shown as mean ± SEM unless otherwise specified. Statistical analysis were performed with Prism 8.0.1 (Graphpad) or MATLAB 2013. Open field and swimming experiments were recorded and analyzed with SMART software (United States). Gait analysis experiment was recorded and analyzed with Gait analysis software (China). Statistical analysis was performed using a one-way analysis of variance (ANOVA) when appropriate. *p* < 0.05 was considered statistically significant. ^∗^*p* < 0.05, ^∗∗^*p* < 0.01, ^∗∗∗^*p* < 0.001, n.s., no significant.

## Results

### rTMS Treatment Partly Reverses the Weight Loss and Muscle Atrophy Caused by SM in Rats

**FIGURE 1:**
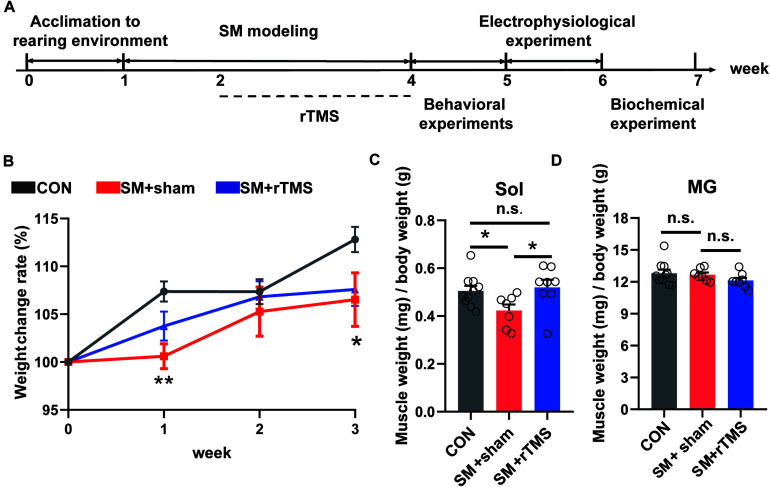
Scheme of the experimental design and effects of rTMS on body weight and muscle weight in SM rats. **(A)** Scheme of the experimental design. The experimental groups were divided into control (CON, *n* = 10), simulated microgravity for 3 weeks with sham stimulation for 2 weeks (SM+sham, *n* = 7) and simulated microgravity for 3 weeks with rTMS for 2 weeks (SM+rTMS, *n* = 8). Behavioral experiments include open field, swimming, and gait analysis experiments. Biochemical experiments include HE staining and western blot experiment. **(B)** The broken-line graph shows the weight change rate of rats during the modeling period. **(C)** Relative mass of Sol muscle. Sol, soleus. **(D)** Relative mass of MG muscle. MG, musculus gastrocnemius. Error bars indicate mean ± SEM. *p < 0.05; **p < 0.01; n.s., no significant, one-way ANOVA.

A schematic of our experimental design is shown in Figure 1A. We firstly observed whether rTMS affect the body weight and muscles of rats related to motor function. The body weight changes during the modeling period summarized in Figure 1B, which illustrated that the relative body weight (body change compared to week 0) in the SM+sham group was significantly lower than the CON group at week 1 and week 3 (Figure 1B, first week, *p* < 0.01; third week, *p* < 0.05), but there was no difference between CON and SM+rTMS (Figure 1B, *p* > 0.05), which suggested that rTMS could restore the weight loss of rats caused by SM.

The relative mass of Sol and MG in hind limbs after the electrophysiological experiment were shown in Figures 1C,D. During SM modeling, the relative mass of Sol muscle reduced in SM+sham compared to controls (42.28 and 50.52%, respectively). However, in the SM+rTMS group, it is 9.90% higher than that in the SM+sham of the relative mass of Sol muscle (*p* < 0.05). And there was no difference between CON and SM+rTMS (Figure 1C, *p* = 0.686). But the relative mass of MG did not differ among groups (Figure 1D, *p* > 0.05). The above results showed that rTMS treatment could partly reverse the weight loss and muscle atrophy caused by SM in rats.

### rTMS Treatment Shortens the Fatigue Period of Rats After SM, but Does Not Improve Myasthenia of Hind Limbs

**FIGURE 2:**
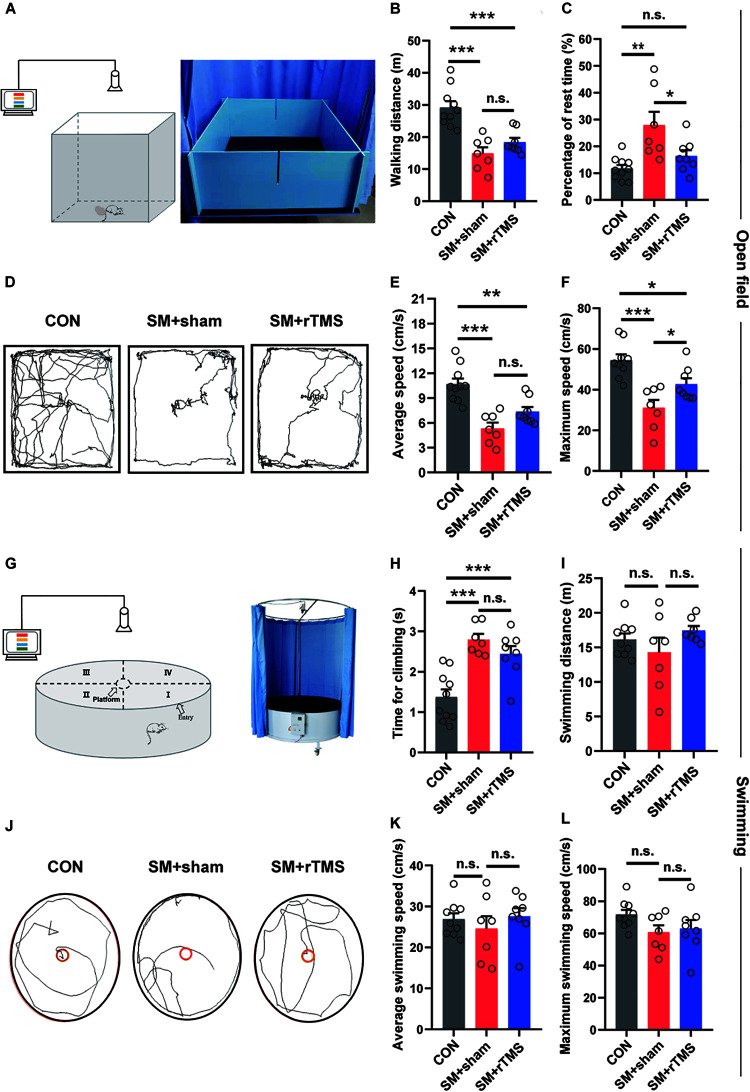
Effects of rTMS on walking and swimming ability of SM rats. **(A,G)** Schematic diagrams of equipment for open field and swimming experiments. **(B)** The walking distance of rats in the open field in 5 min. **(C)** The time of rats rested in the open field within 5 min. **(D,J)** The trajectories of rats among groups in the open field and swimming process, respectively. **(E,F)** The average and maximum speed of rats walking in the open field, respectively. **(H)** Average time for climbing on the platform of three groups, since forelimbs reaching the platform to all the hind limbs on the platform. **(I)** The distance of rats swimming in the tank in 1 min. **(K,L)** The average and maximum speed of rats swimming in the tank, respectively. Error bars indicate mean ± SEM. *p < 0.05; **p < 0.01; ***p < 0.001; n.s., no significant, one-way ANOVA.

Next, we measured the walking and swimming ability of the rats by observing the performance in an open field (Figures 2A,D). We found that when compared to CON, SM+sham displayed a significant reduction in walking distance (CON: 29.21 ± 1.94 m; SM+sham: 14.87 ± 1.94 m; *p* < 0.001), the average speed (CON: 10.68 ± 0.68 cm/s; SM+sham: 5.33 ± 0.70 cm/s; *p* < 0.001) and maximum speed (CON: 54.44 ± 2.94 cm/s; SM+sham: 31.01 ± 3.94 cm/s; *p* < 0.001) (Figures 2B,E,F). And for the SM+rTMS, these parameters still decreased significantly compared with the CON (walking distance: 18.40 ± 1.30 cm; average speed: 7.37 ± 0.56 cm/s; maximum speed: 42.70 ± 2.92 cm/s). Besides, SM+sham rats had a significant increase in the rest time compared to controls (CON: 11.77 ± 1.32 s; SM+sham: 27.88 ± 4.99 s; *p* < 0.01), however for the SM+rTMS, the rest time was significantly decreased as compared to the SM+sham rats, which approached the rest time in controls (SM+rTMS: 16.38±2.17 s, Figure 2C, *p* > 0.05).

Next we evaluated differences in groups based on the swimming behavior of the rats (Figures 2G,J). As shown in Figures 2I,K,L, SM did not induce major disturbances in the swimming behavior (*p* > 0.05). SM significantly increased the time for climbing onto the platform (CON: 1.37 ± 0.19 s; SM+sham: 2.79 ± 0.14 s; Figure 2H, *p* < 0.001), which was mainly associated with the function of the hind limbs, however rTMS had no effect on the procedure (SM+rTMS: 2.44 ± 0.20 s, *p* < 0.001). These results indicated that SM was associated with fatigue and myasthenia in rats. With the treatment of rTMS, the fatigue period of the rats became shorter, but the myasthenia of hind limbs caused by SM was not improved.

### rTMS Treatment Can Improve the Contralateral Limb Imbalance Caused by SM to Some Extent

**FIGURE 3:**
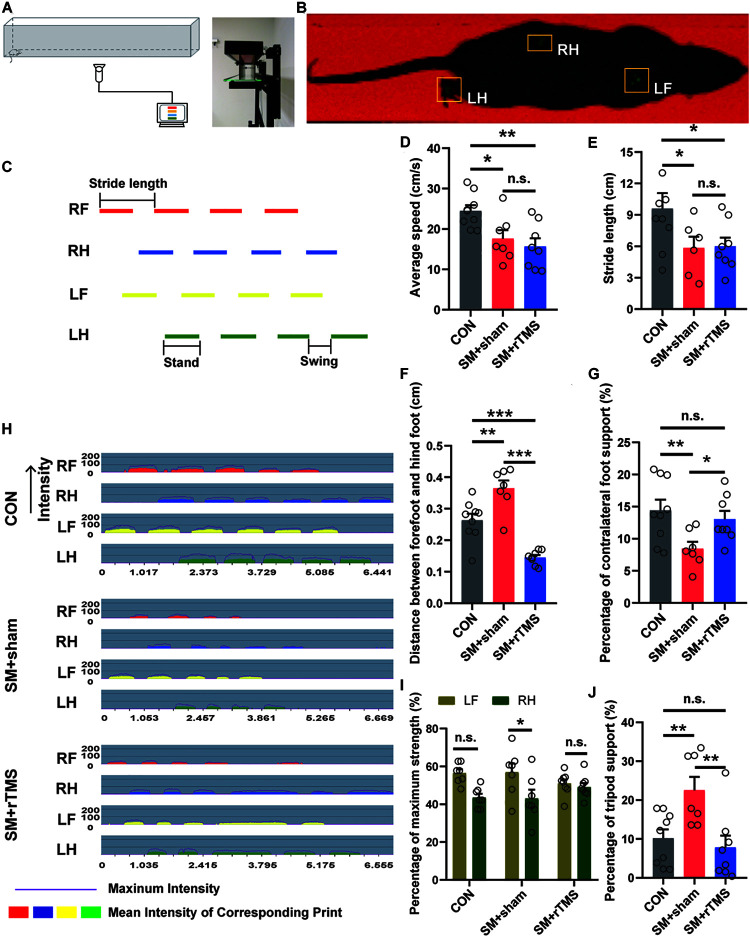
Effect of rTMS on the gait of SM rats. **(A)** Diagram of experimental equipment for gait test. **(B)** The actual trajectory of the rat walking. RF, right front; RH, right hind; LF, left front; LH, left hind. **(C)** The graph of the time step sequence, which reflects the sequence of different feet walking on the track. Stride length, stand, and swing definitions are shown in the figure. **(D)** The average speed of the rats walking on the track. **(E)** The length of rats’ strides during walking. **(F)** Distance between forefoot and hindfoot of rats. **(G,J)** The percentage of time spent on contralateral foot or tripod support of rats. **(H)** The diagram of pressure-time of the each foot of rat, which reflects the pressure of each foot contacting with the ground. The intensity ranges from 0 to 255 MPa. **(I)** The maximum strength of contact between the contralateral feet and the ground (LF, left front foot; RH, right hind foot). *p < 0.05; **p < 0.01, ***p < 0.001; n.s., no significant, one-way ANOVA. Error bars, SEM. See also Supplementary Figure 1.

Previously, we used the open field experiment to measure the locomotor activity in an open field arena (Kraeuter et al., 2019). In order to eliminate the influence of mood change on gait behaviors in rats, we adopted more professional equipment to examine the gait of rats (Figures3A,B). It was found that the walking speed of the rats on the track was consistent with the experimental results in the open field (CON: 24.49 ± 1.40 cm/s; SM+sham: 17.61 ± 2.11 cm/s; SM+rTMS: 15.70 ± 2.00 cm/s; Figure 3D). When observing the stride length of rats (Figure 3C), it was found that rTMS had no significant effect on the decrease of the stride length caused by SM (CON: 9.60 ± 1.47 cm; SM+sham: 5.84 ± 1.08 cm; SM+rTMS: 5.60 ± 0.83 cm; Figure 3E). While it was worth noting that SM caused a increase of step spacing, indicating that the hind limbs were overextended, and rTMS could reverse this change (CON: 0.26 ± 0.02 cm; SM+sham: 0.36 ± 0.03 cm; SM+rTMS: 0.15 ± 0.01 cm; Figure 3F, CON vs SM+sham, *p* < 0.01; CON vs SM+rTMS, *p* < 0.001). When comparing the time support percentage of different feet, SM or rTMS treatment did not affect the single foot and ipsilateral feet support percentage (Supplementary Figures 1A,B, *p* > 0.05), but significantly decreased the support time of the contralateral feet (CON: 14.43 ± 1.66%; SM+sham: 8.44 ± 1.07%; Figure 3G, *p* < 0.01). Further observation of the strength revealed that SM caused an uneven distribution of the strength of the contralateral feet (Figure 3I, *p* < 0.05), and rTMS could improve these effects to a certain extent. Figure 3H showed the time-dependent variation of the pressure of each foot contacting with the ground, with the intensity ranging from 0 to 255 MPa. The support time of the tripod feet was significantly increased in the SM+sham group (CON: 10.19 ± 2.24%; SM+sham: 22.55 ± 3.43%; Figure 3J, *p* < 0.01), which recovered to a normal level with rTMS treatment (SM+rTMS: 7.80 ± 3.10%). These results suggested that rTMS can restore the contralateral limb imbalance caused by SM during the movement of rats.

**FIGURE 4:**
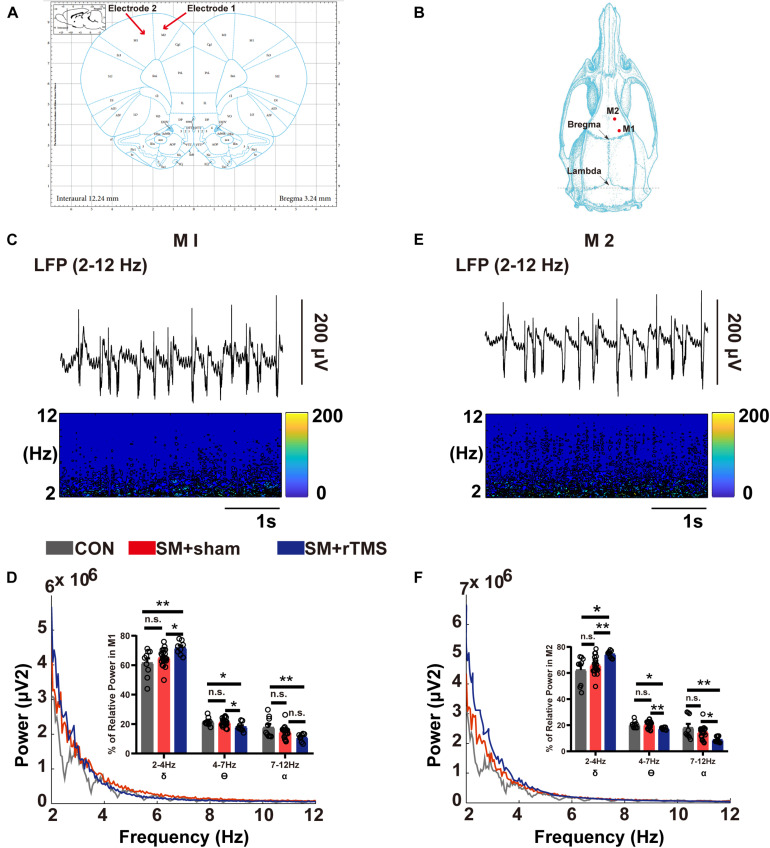
Brain electrical activity of M1 and M2 regions in SM rats. **(A)** The M1 and M2 regions of the motor cortex are shown in the brain atlas. **(B)** Diagram of the position coordinates of craniotomies during surgical procedure. **(C,E)** The corresponding spectrogram of LFP activity, indicating changes in power at 2–12 Hz in M1 and M2 regions. **(D,F)** Results of LFP activities in the M1 and M2 regions. Power spectra showed a mean level of LFP activities from all the rats in CON (gray line), SM+sham (red line) and SM+rTMS (blue line). The bar graphs represent the mean level of relative LFP power in the M1 and M2 within a series of frequency ranges: 2–4, 4–7, and 7–12 Hz. *p < 0.05; **p < 0.01; n.s., no significant, one-way ANOVA. Error bars, SEM.

### rTMS Can Influence the Neural Oscillation by Changing the Energy Distribution in Different Frequency Bands in the M1 and M2 Regions

We recorded the electrophysiological signals from M1 and M2 areas of the rats (Figures 4A,B) and preliminarily screened the collected LFP signals and further analyzed with MATLAB software. In M1, Figures 4C,D showed that compared to control rats, the power of LFP in low-frequency band in rats after rTMS treatment was enhanced while suppressed in high-frequency band. To further quantify and precisely evaluate the effect of rTMS treatment on the power spectra of M1, the relative LFP power in three bands were computed in the signals (the bar graph in Figure 4D). ANOVA analysis showed a significant effect in the δ (2–4 Hz), θ (4–7 Hz), and α (7–12 Hz) bands. Compared to control group, the group treated with rTMS showed higher relative powers of δ (61.57 ± 3.07 vs 71.77 ± 1.60, *p* = 0.002) and lower relative powers of θ (21.61 ± 0.92 vs 18.10 ± 1.08, *p* = 0.01), and α (17.80 ± 2.30 vs 11.02 ± 0.92, *p* = 0.004). In M2, the relative LFP powers had similar results to those observed in M1 (Figures 4E,F). Compared to control rats, the rats treated with rTMS showed higher relative power of δ (62.32 ± 3.65 vs 74.50 ± 0.97, *p* = 0.002), lower relative power of θ (20.53 ± 0.88 vs 17.53 ± 0.34, *p* = 0.015), and α (18.07 ± 2.93 vs 8.84 ± 0.86, *p* = 0.002). The results of rTMS treatment showed that rTMS can influence the neural oscillation by changing the energy distribution in different frequency bands in M1 and M2 regions.

### rTMS Treatment Can Affect the Apoptosis of Nerve Cells

**FIGURE 5:**
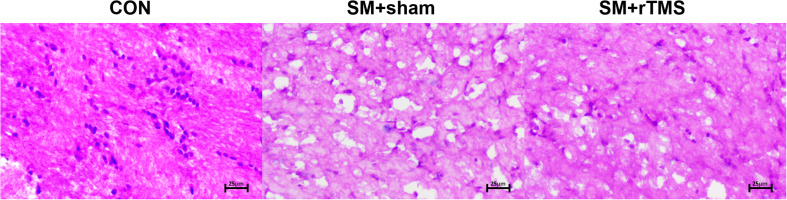
Schematic results of HE staining of motor cortex. Morphological changes of nerve cells in motor cortex. The sections of tissue were HE stained. In the schematic diagram, the shape difference and rupture degree of different cells in different groups can be observed. Pictures were all at 20× magnification.

Then we examined whether SM and rTMS treatment had any effects on the morphology of neurons. The observation was as follows: in the motor cortex, the neurons in the CON group were closely arranged, while in the SM+sham group there were notable tissue changes characterized by fewer cells and scattered arrangement. In the rTMS group, the cells were occasionally ruptured, but the tissues were intact (as shown in Figure 5). Summary of the above showed that rTMS had a certain effect on the morphological changes of the above nerve cells, which may be one of the reasons why it could influence the neural oscillation.

### rTMS Treatment Activates mTOR in the Motor Cortex

**FIGURE 6:**
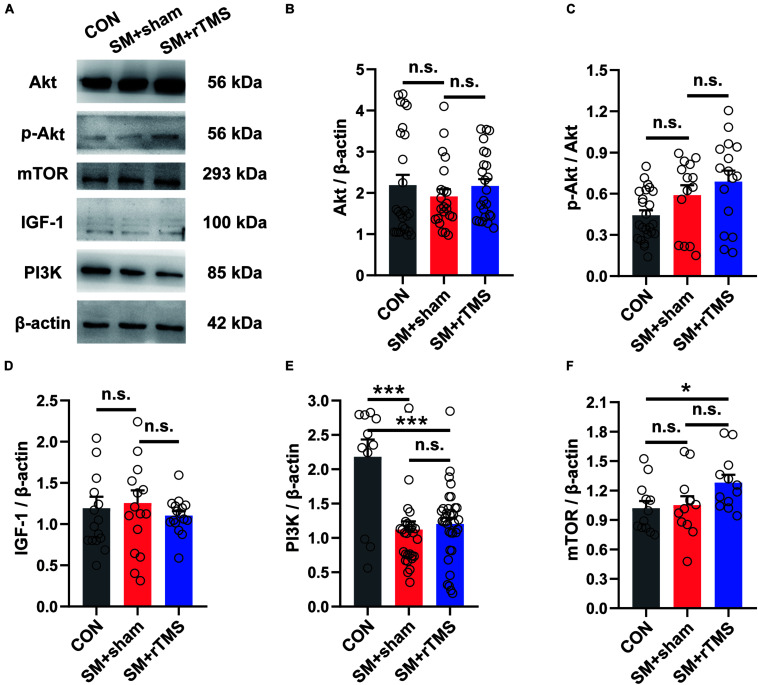
Effects of rTMS on the IGF-1-PI3K-Akt-mTOR signaling pathway in the motor cortex. **(A)** Representative western blot bands using total protein extracts from the motor cortex. **(B–F)** Quantification of protein expression levels in the motor cortex among the CON, SM+sham, and SM+rTMS group (*n* > 10, from five rats in each group). *p < 0.05; ***p < 0.001; n.s., no significant, one-way ANOVA. All values are mean ± SEM.

Human studies have demonstrated the pivotal role of mTOR in exercise-dependent cortical neural remodeling, which helps to improve the acquisition of motor skills (Chen K. et al., 2019). So after the electrophysiology recording, we then investigated the expression of IGF-1, Akt and its phosphorylated protein in the motor cortex (Figure 6A). The results showed that SM and rTMS had no significant effect on their expression (Figures 6B–D, *p* > 0.05). Further, the expression of PI3K protein was decreased by SM (CON: 2.18 ± 0.25; SM+sham: 1.12 ± 0.12; *p* < 0.001), which was not impacted by rTMS (SM+rTMS: 1.20 ± 0.09; Figure 6E). We also found elevated mTOR in the motor cortex after rTMS treatment (CON: 1.02±0.07; SM+sham: 1.05±0.09; SM+rTMS: 1.28 ± 0.08; Figure 6F, CON vs SM+rTMS, *p* = 0.031), indicating the activation of mTOR by rTMS. These data collectively demonstrated rTMS-induced mTOR activation in the motor cortex, but it did not affect the downregulation of PI3K caused by SM.

## Discussion

Despite dozens of studies reporting that the beneficial effects of rTMS in improving dyskinesia in different models of rats including spinal cord injury, Parkinson diseases and cerebral ischemia (Ba et al., 2016; Li et al., 2016; Arfani et al., 2017; Petrosyan et al., 2017), it is understood that the underlying causes of dyskinesia in above diseases are different from microgravity in causing gait disorders in astronauts. So the underlying mechanism of rTMS acting on gait disorders caused by SM is still less known. Our current study provides the first evidence showing that rTMS treatment can improve the gait disorders caused by SM to some extent and clarifies its mechanism from different perspectives, including behavioral experiments (open field, swimming, and gait analysis), electrophysiological experiment and biochemical tests (HE staining and western blot).

The first finding is that rTMS can potentially reverse SM-induced weight loss in rats and improve muscle atrophy, however the changes in body weight may have been caused by modeling effects on feeding and excretion in rats (Pan et al., 2019). As for sport-related hindlimb muscles, why did SM cause the Sol to atrophy, but not the MG? This may be because SM causes a shift in muscle type from slow (I type fiber) to fast (II type fiber) (Xu et al., 2015). Sol is a type I fiber muscle (Faulkner et al., 2018), and additionally it is possible that fluid loss demonstrated by Sol atrophy is caused by head displacement, which is consistent with previous results (Gumucio and Mendias, 2013). Reversal of Sol atrophy associated rTMS treatment may be related to the expression of some motor-related proteins in Sol, but more research is needed to understand this relationship.

The second finding is rTMS impacts motor abilities after SM, improving motor behaviors to some extent. Gait analysis is a commonly used method in the study of the behavior of mice, which could detect many indicators such as footprint area, stride length, footprint support, footprint strength, and so on (Hruska et al., 1979; Deumens et al., 2007). Based on this, one important result was that rTMS largely mitigated SM-induced shorter supporting time for the contralateral feet and longer supporting time for the tripod. Similar results were obtained for the strength of the contralateral foot (Figure 3I and Supplementary Figure 1C). These results indicate that rTMS can improve the imbalance of the contralateral limb caused by SM during the movement of rats, however it needs to be noted that there is still myasthenia in the hind limbs that was not alleviated.

The motor cortex is essential for the execution of motor information, and M1 and M2 are the most advanced centers of body control (Byblow et al., 2007). The M1 is responsible for the coding of movement (Bryszewski et al., 2012), the M2 is responsible for the integration of spatial information toward the updating of planned movements, which is further projected to the M1, brainstem, spinal cord, and other regions involved in motion control (Olson et al., 2020). Therefore, LFP signals in M1 and M2 regions were recorded in this study to explore the effect of rTMS treatment on neural oscillations in motor cortex. We have two main conclusions from the results: (1) It was found for the first time that SM did not affect LFP activity (2–12 Hz), which had not been noticed before. In other models, such as Parkinson’s rats, the power of motor cortex is inhibited at 1–4 Hz (Geng et al., 2016a), and the interaction between motor cortex and pedunculopontine nucleus in the range of θ (4–7 Hz) and α (7–12 Hz) is enhanced (Brazhnik et al., 2012). These abnormal neural oscillation activities are closely related to the dyskinesia symptoms of Parkinson’s rats, such as abnormal gait and unstable posture (Park et al., 2014; Geng et al., 2016b). In our study, we did not find a similar change indicating the specificity of different conditions. To explain why the abnormal behavior can be observed in SM, we give two possible mechanisms. Firstly, SM may affect the high-frequency LFP signal of motor cortex, which has been suggested in the previous study: the symptoms of gait disorders in SM rats are always related to the neural oscillations in the motor cortex with the peak frequency of LFP ∼80 Hz (Brys et al., 2018). However, we did not detect in our study due to the limitations of the experimental conditions which can be further recorded under the awake state (Belluscio et al., 2003; Rolland et al., 2007; Wang et al., 2015). So SM may cause the change of neural rhythm in its downstream neural network, which is manifested as the disturbance of motor behavior, but not the change of neural oscillation in motor cortex.

(2) The effects of rTMS on M1 and M2 of SM rats were similar, both of which increased the energy of δ (2–4 Hz) and decreased in θ (4–7 Hz) and α (7–12 Hz). A previous study pointed that rTMS, as a neural regulatory technique, can modulate cortical brain rhythms, especially low-frequency oscillations (Noh, 2012). So we speculate that rTMS could regulate the energy distribution of different frequency bands (δ, θ, and α) of motor cortex, especially by enhancing the energy of δ (2–4 Hz) frequency band. This has proved the hypothesis that we put forward earlier.

The above results suggested that rTMS may improve gait disorders by regulating neural oscillations in motor cortex. But the specific molecular mechanism remained to be further discovered. We examined the expression of IGF-1-PI3K-Akt-mTOR pathway in the motor cortex. IGF-1 has been proved to increase neurotransmitter release and discharge of pyramidal neurons by promoting Ca^2+^ entry into the motor cortex, and excitability of sensorimotor cortex neurons (Nishijima et al., 2010; Ding et al., 2016). PI3K protein is then activated by IGF-1, which induces the phosphorylation of Akt and controls the activation of two targets related to protein synthesis, one of which is mTOR (Tang et al., 2014). In this study, we did not observe the change of IGF-1 and Akt proteins in the motor cortex after SM modeling. This is inconsistent with previous results in which they were found decreased after a 14-day period of SM (Chen B. et al., 2019). The inconsistency may be due to the length of modeling time (Nishijima et al., 2010; Mysoet et al., 2017). However, it has been reported that the activation of mTOR can promote axonal regeneration of corticospinal neurons, and then enhance neuronal activity (Zareen et al., 2018). Our results suggest that rTMS may enhance the neuronal activity of the motor cortex through the activation of mTOR, leading to the improvement of gait disorders. So mTOR can be an attractive therapeutic target and deserves further investigations.

## Conclusion

Combining our results with previous studies, it was found that there is a progressive reduction in the ability of motor cortex to produce muscle forces under SM conditions. Processes within the nervous system, as well as within the muscles both contribute to gait disorders (Pannérec et al., 2016). Our results indicated that the regulation at high level of the nervous system could improve gait ability and fatigue induced by SM. The mechanism was associated with the regulation of the energy distribution of different frequency bands (δ, θ, and α) of motor cortex, especially by enhancing the energy of δ (2–4 Hz) frequency band, as well as the activation of mTOR protein by rTMS, which could enhance the control of nervous system on hindlimb muscle, ultimately achieving the improvement in gait behaviors. However, during rTMS treatment, the relative contribution of nerve and muscle factors in motor improvement has not been thoroughly understood (Clark et al., 2006), and further studies are still needed.

## Data Availability Statement

The raw data supporting the conclusions of this article will be made available by the authors, without undue reservation.

## Ethics Statement

The animal study was reviewed and approved by the Animal Management Rules of the Ministry of Health of the People’s Republic of China and the local Ethical Committee of the Tianjin University.

## Author Contributions

JY conceived the idea, supervised the work, and participated in the revision of the manuscript. RL involved in designed the study, completed the experimental operation and subsequent data processing, and wrote the manuscript. LW and other authors involved in planning during the early phase of this project and follow-up to assist the experiment to further improve. All authors reviewed and contributed to the final manuscript.

## Conflict of Interest

The authors declare that the research was conducted in the absence of any commercial or financial relationships that could be construed as a potential conflict of interest.

## Supplementary Material

Supplementary Figure 1 Effects of rTMS on gait in SM rats. **(A,B)** The percentage of time spent on single and ipsilateral feet support in rats. **(C)** The maximum strength of contact between the contralateral feet and the ground (RF, right front foot; LH, left hind foot). **p* < 0.05; n.s., no significant, one-way ANOVA. Error bars, SEM.
