# Supplementary material for: Repetitive Transcranial Magnetic Stimulation (rTMS) Improves the Gait Disorders of Rats Under Simulated Microgravity Conditions Associated With the Regulation of Motor Cortex
Source: Front Physiol. 2021 Feb 4;12:587515. doi: 10.3389/fphys.2021.587515 (PMC7890125; doi:10.3389/fphys.2021.587515)
Supplement: Supplementary Figure 1 — Effects of rTMS on gait in SM rats. (A,B) The percentage of time spent on single and ipsilateral feet support in rats. (C) The maximum strength of contact between the contralateral feet and the ground (RF, right front foot; LH, left hind foot). *p < 0.05; n.s., no significant, one-way ANOVA. Error bars, SEM. [file Data_Sheet_1.PDF]

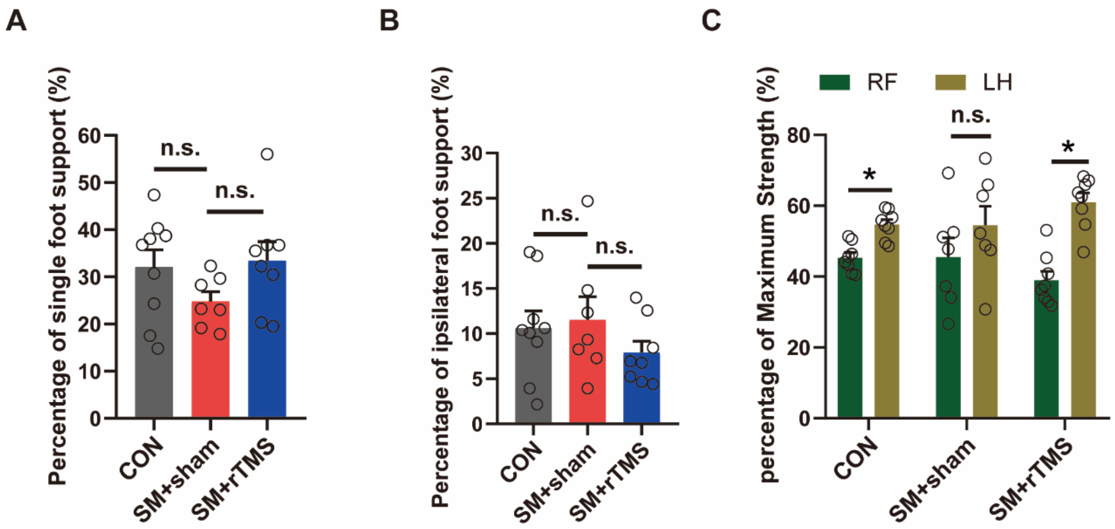

A
B
C
Percentage of single foot support (%)
n.s.
n.s.
CON
SM+sham
SM+rTMS
Percentage of ipsilateral foot support (%)
RF
LH
percentage of Maximum Strength (%)
*

Fig. S1. Effects of rTMS on gait in SM rats. (A, B) The percentage of time spent on single and ipsilateral feet support in rats. **(C)** The maximum strength of contact between the contralateral feet and the ground (RF, right front foot; LH, left hind foot). *$p<0.05$; **$p<0.01$; *n.s.*, no significant, one-way ANOVA. Error bars, SEM.
